# Urban environment predisposes dogs and their owners to allergic symptoms

**DOI:** 10.1038/s41598-018-19953-3

**Published:** 2018-01-25

**Authors:** Emma Hakanen, Jenni Lehtimäki, Elina Salmela, Katriina Tiira, Johanna Anturaniemi, Anna Hielm-Björkman, Lasse Ruokolainen, Hannes Lohi

**Affiliations:** 1 Department of Biosciences, University of Helsinki, PL 65 FI-00014, Helsinki, Finland; 2 Research Programs Unit, Molecular Neurology, and Department of Veterinary Biosciences, University of Helsinki, PL 63 FI-00014, Helsinki, Finland; 3 The Folkhälsan Institute of Genetics, PL 63 FI-00014, Helsinki, Finland; 4 Department of Equine and Small Animal Medicine, University of Helsinki, PL 57, FI-00014, Helsinki, Finland

## Abstract

Our companion-animals, dogs, suffer increasingly from non-communicable diseases, analogous to those common in humans, such as allergic manifestations. In humans, living in rural environments is associated with lower risk of allergic diseases. Our aim was to explore whether a similar pattern can be found in dogs, using a nation-wide survey in Finland (*n* = 5722). We characterised the land-use around dog’s home at the time of birth as well as around its current home, and described several lifestyle factors. The severity of owner-reported allergic symptoms in dogs was estimated with a comprehensive set of questions, developed by experts of canine dermatology. Also, the prevalence of diagnosed allergies in dog owners was recorded. The results indicate that allergic symptoms are more prevalent in urban environments both in dog owners and in dogs (accounting the effect of dog breed). Several factors related to rural living, such as bigger family size and regular contact with farm animals and other pets, were also protective against allergic symptoms in dogs. Interestingly, allergic dogs were more likely to have allergic owners than healthy dogs were. Therefore, we suggest that the mutual presence of allergic symptoms in both species indicates common underlying causal factors of allergic diseases.

## Introduction

Western diet^1^ and urbanisation^2^ are known to contribute to the increasing epidemic of inflammatory disorders in humans. These factors are thought to be mediated via the host microbiota^3^, and can be even interactive in affecting host microbiota and physiology^4^. Reduced contact with environmental microbial diversity, due to altered living environment, diet, and overall lifestyle, provides less of the vital signalling a developing immune system requires^5–8^ as suggested in the biodiversity hypothesis^9^. Different environments differ in their microbial communities^10^, and thus the living environment has the potential to influence the composition of human microbiota^11–13^. In turn, bacteria differ in their immuno-regulatory potential^14–16^, meaning that the sort of bacterial exposure an individual receives is not necessarily trivial. Interestingly, inflammatory disorders have also increased in our companion animals, dogs^17^, and many of these disorders are analogous to human diseases^18^. For example, canine atopic dermatitis (CAD) bears numerous similarities to human atopic dermatitis^19,20^. While studies on allergic diseases in dogs in association with environment and lifestyle are limited, previous work suggests that contact with rural environments can be protective in dogs^17,21^, as suggested for humans^13^.

Large-scale epidemiological studies are tedious and time-consuming to conduct in humans, due to, e.g., laborious preparations and difficulty to find participants. Moreover, the results are not straightforward to interpret due to multiple confounding factors arising from the complexities of human life. Dogs, on the other hand, usually live much simpler and shorter lives, and could hence serve as a model animal in understanding shared diseases both in dogs and humans more mechanistically^22^. Also, dogs have higher genomic similarity to humans^23^ (~95%) than mice do^24^ (~90%). The relatively large size of dogs makes them also physiologically and clinically more similar to humans than the mouse model. Regarding heredity of inflammatory diseases, the pedigrees and health examination results are well-recorded by kennel clubs or equivalent organizations in many countries, including Finland. Dogs are also exposed to the same living environment and lifestyle features as their owners—supported by studies showing that dogs and their owners share their microbiota^25,26^, which makes dogs a relevant model in environmental health studies.

Dogs suffer from atopic dermatitis and food allergies similarly to humans, whereas allergic symptoms associated with the lower respiratory tract are basically absent in dogs^20^. CAD is a genetically predisposed inflammatory and pruritic allergic skin disease with characteristic clinical features^27^. It is associated with elevated allergen-specific immunoglobulin E antibodies (IgE; a common marker of allergies in humans)^28^ but the exact role played by IgE and other antibodies in the pathogenesis of atopic dermatitis is still undetermined^29^. Therefore, there is no definitive diagnostic test for CAD, and consequently the disease is diagnosed by eliminating other pruritic skin disease and by the fulfilling of certain clinical and historical criteria^30,31^. The other common allergic disease in dogs is food allergy (FA), which can be separated from non-immune mediated reactions to food, such as food poisoning and metabolic reactions^32,33^. The symptoms in CAD and FA are partly overlapping. Consequently, these diseases are difficult to distinguish^33^.

The objective of this study was to analyse the environmental risk factors of allergy-related symptoms in dogs, using a large survey data set. We tested whether previously reported associations between the living environment and allergic diseases in humans can be generalized to dogs. We also tested the concordance of the occurrence of allergy in dogs and their owners.

## Results

As breed is an important factor in dog allergies, we created two subsets from the data, in attempt to control for the effect of breed. These subsets were *Allergy-tolerant breeds* and *Common breeds*, from which we excluded those breeds defined as allergy-prone in previous research (Table S4). For the *Common breeds*, we selected only the five most represented breeds in the data.

### Rural living environments are inversely associated with the risk of allergic symptoms in dogs

**Figure 1:**
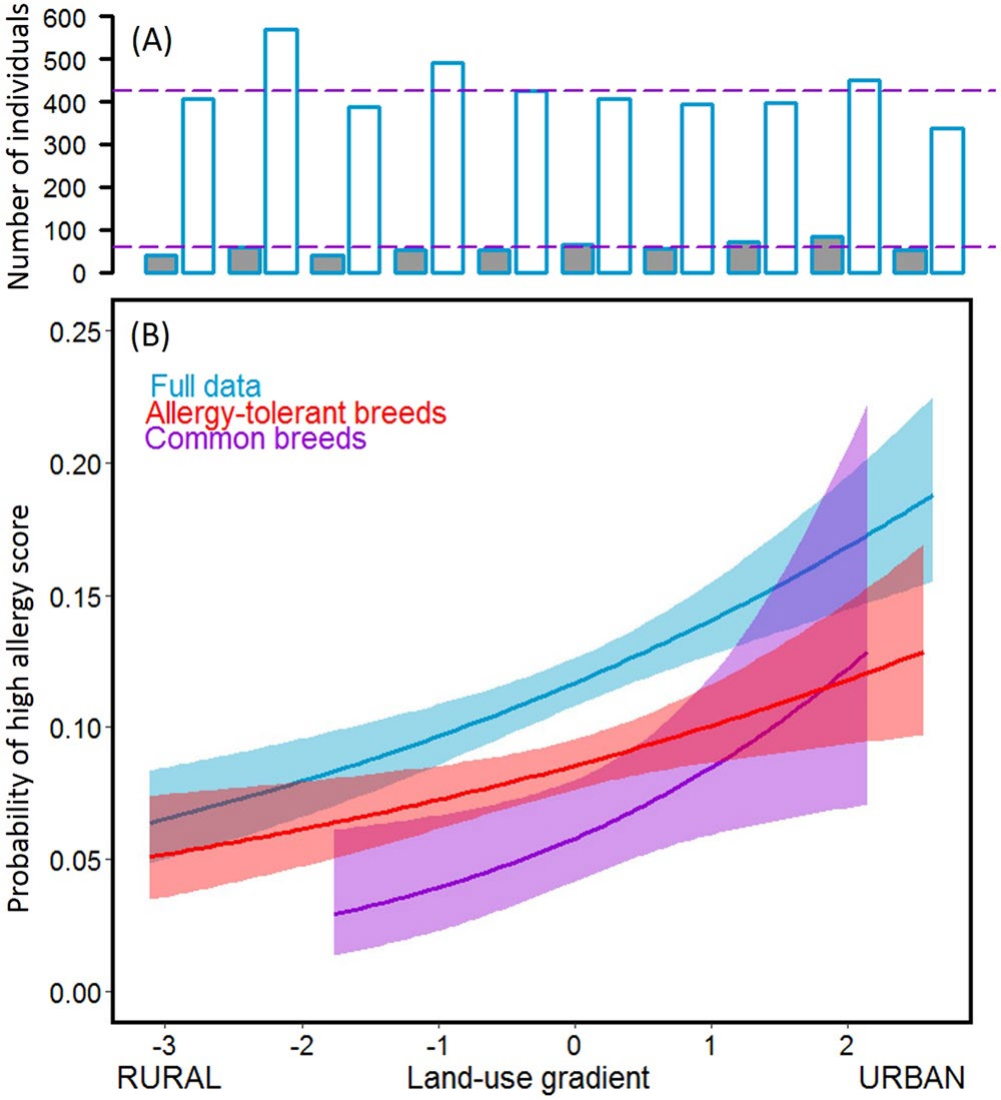
Allergic symptoms are more common in urban environments in dogs. Upper panel (A) shows the number of individuals with a high allergy score (dark grey) and a low allergy score (white) along the land-use gradient in the full data. The purple lines indicate the mean size of high allergy score and low allergy score groups. The proportion of built environment increases along the land-use gradient (x-axis). Lower panel (B) shows the probability of high allergy score along the land-use gradient. The high allergy score is common in urban environments in all subsets i.e. the differing occurrence of breeds in rural and urban environments does not confound the result.

Most dogs were born in rural areas (*Full data*: 72%, *n* = 3386; *Allergy-tolerant*: 74%, *n* = 2351; *Common breeds*: 77%, *n* = 447). Approximately half of the dogs were currently living in rural and in urban environments (*Full data*: 52%, *n* = 2945; *Allergy-tolerant*: 53%, *n* = 2051; *Common breeds*: 46%, *n* = 338 of the dogs in rural environments). Based on our questionnaire data, we assigned each dog an allergy score (see Methods); a numeric value indicating the severity of allergic symptoms. When concentrating only on those dogs with either low (mild allergic symptoms) or high (severe allergic symptoms) allergy score, rural environments in the current living area were associated with lower risk of high allergy score in each subset (Fig. 1, Table 1). However, no association could be found in the case of the birth environment. Similar patterns in each subset suggests that the differing prevalence of allergy-prone breeds in urban and rural environments did not explain the result.

**Table 1:** Logistic regression on the risk of high allergy score (based on owner-reported allergic symptoms) against environmental land-use around the dog’s home at birth and the current home in all subsets. When current living environment becomes more urban, odds to become allergic are higher. Odds Ratio, OR = exp(estimate).

Environment	Subset	*n*_*low score*_	*n*_*high score*_	OR	*P*
Birth home	*Full data*	3604	422	1.04	0.484
	*Allergy-tolerant breeds*	2565	209	1.01	0.906
	*Common breeds*	477	24	1.17	0.444
Current home	*Full data*	4292	572	1.24	8.3e–07
	*Allergy-tolerant breeds*	3045	284	1.20	0.003
	*Common breeds*	584	39	1.51	0.014

### Nature-oriented lifestyle provides an additional barrier against canine allergy

**Figure 2:**
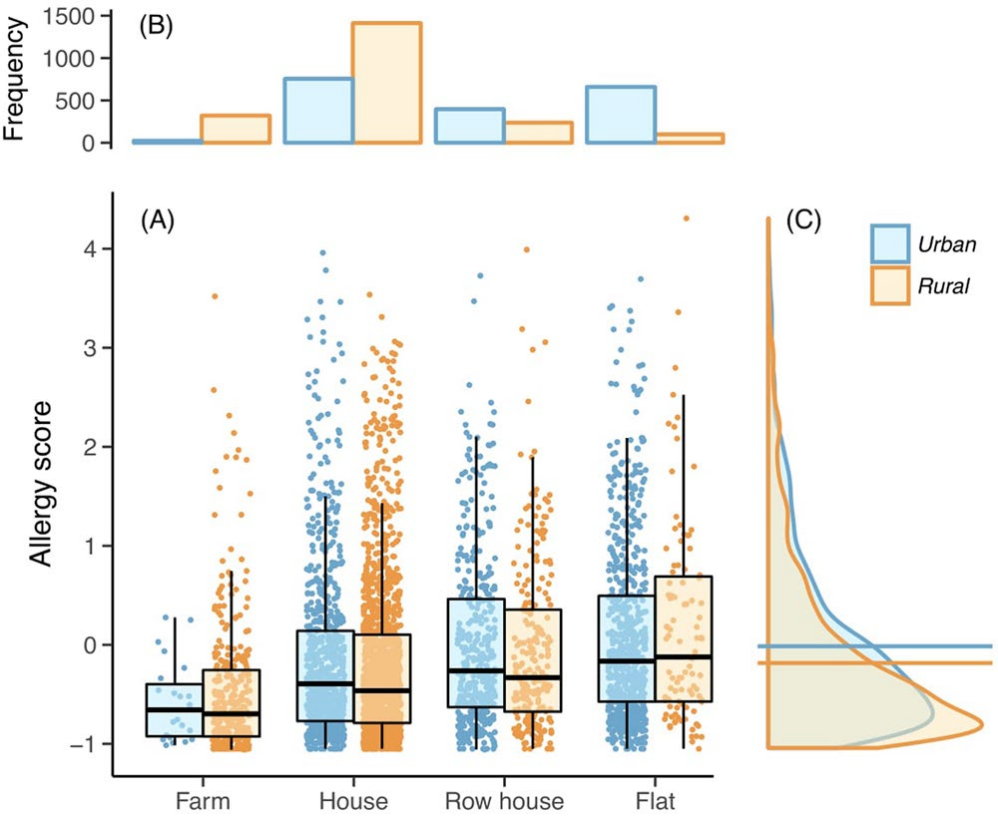
Housing type in relation to allergy score in allergy-tolerant breeds. (**A**) The type of living apartment was associated with the allergy score (*P* < 0.0001, based on generalized least squares, assuming group-specific residual variance). While the pattern was repeated for both urban (blue) and rural (orange) environments (and overall the allergy score was higher in urban environments as shown in (**C**); *P* = 1.3e^−9^), the type of the apartment was also related to the living environment—this is shown in panel (**B**) (*χ*^2^ = 901.4, *P* < 2.2e^−16^), and thus the effect of these is hard to separate. (**B**) The frequency of different housing types across urban and rural environments. (**C**) The distribution of allergy score in urban and rural individuals, across all housing types. Horizontal lines indicate mean values for each distribution showing that the mean allergy score is higher in urban than in rural individuals. In Fig. 2B,C the redundant axes have been omitted.

While the composition of residential land-use can be easily quantified, its role in health can be confounded by several factors. Several factors that relate rural lifestyle, such as dog living in outdoor conditions and having regular contact with farm animals, were associated with allergy protection (Table 2). Additionally, while the type of living apartment was significantly associated with the allergy score (Fig. 2A), house type was also significantly associated with the residential area (*χ*^2^ = 901, 41, *P* = 2.2e–16; Fig. 2B). Thus, it is not straightforward to interpret the higher allergy score associated with rural areas in comparison to urban areas (Fig. 2C).

**Table 2:** The effect of several factors related to rural living in association with allergy score in dogs. Trinominal factors were tested with Kruskal-Wallis test and binominal factors with Mann-Whitney U-Test against allergy score. The protective factors are underlined. Visiting city areas and farm animal contact happened at the age of 4 weeks to 6 months.

Protective effect	*Full data*			*Allergy-tolerant breeds*			*Common breeds*		
	*n*	*Z/Χ* ^2^	*P*	*n*	*Z/Χ* ^2^	*P*	*n*	*Z/Χ* ^2^	*P*
Living condition (outside, both, inside)	2897	131.4	2.9e-29	2014	84.10	5.4e-19	330	15.2	0.00049
Agriculture (yes, no)	2921	3.5	0.00047	2030	−3293	0.001	335	−1.38	0.169
Visiting city areas (frequent, seldom, never)	2716	18.9	0.000079	1898	15.98	0.00034	316	12.64	0.002
Farm animal contact (frequent, seldom, never)	2622	9.96	0.007	1830	4.81	0.09	301	2.49	0.288
Other pets in household (yes, no)	2945	7.85	4.10e-15	2051	−6.03	1.6e-9	338	−4.20	0.000026
Farm animals in household (yes, no)	2945	4.81	0.000002	2051	−4.06	0.000049	308	−1.64	0.1

To capture the effect of confounding factors, we constructed a single model, including the current residential land-use and several factors previously associated with allergies in human studies. Using a subset of *Allergy-tolerant breeds*, we further excluded 192 breeds that had less than 50 individuals, such that the effect of breed could be more easily controlled in the model. We included three independent environmental factors (outdoor exposure, outdoor exercise, and agricultural lifestyle; see Methods), house type, and the land-use gradient. When analyzed alone (together with the random effects of breed and sex), land-use was a significant predictor of allergy score (*n* = 1658, *P* = 0.00020). However, together with the other explanatory variables it was no longer significant (Table 3), whereas outdoor exposure, outdoor exercise and house type were all significant (Table 3).

**Table 3:** Model for several factors in association to allergy score. Breed and sex were set as random factors (sex nested in breed). The outdoor exposure, outdoor exercise and house type remained significant predictors of allergy score when factors mentioned in table were included in the same analysis.

Predictor	df	MS	*F*	*P*
Outdoor exposure	1	0.61	0.96	0.00072
Outdoor exercise	1	29.37	46.57	5.6e–6
Agricultural lifestyle	1	0.14	0.22	0.83
House type	4	2.76	4.38	0.0032
Land use	1	0.011	0.017	0.90

Moreover, a big (human) family size seemed to be protective, as the allergy score in dogs was lower in families with three children or more, in comparison to families with only 1–2 children. The environmental land-use was controlled in this analysis due to differences in family-size between rural and urban areas (*Allergy-tolerant breeds:* Regression df = 2, MS = 11.76, *F* = 16.41, *P* = 9.91e–8, predictors family size *t* = -2.74, *P* = 0.06 and land use *t* = 4.89, *P* = 1e–6).

### Canine allergy is associated with allergy in their owners

In our data, we could test the effect of the current residential environment on allergy prevalence among dog owners. In agreement with the above results on dogs, allergies in dog owners tended to be more common in urban areas. This effect could be seen both in the adults and children in the family (Table 4). Interestingly, allergy in the dog and its owner occurred concurrently, even when controlling for the land-use effect. If a dog was allergic, the owner was more likely to be allergic as well (Logistic regression for children in a family *n* = 1173, OR = 0.63, *P* = 0.026 and land-use OR = 1.24, *P* = 0.048; for adults in a family *n* = 5722, OR = 0.66, *P* = 3e–6 and land-use OR = 1.23, *P* = 3e–6). This effect could also be seen in the subsets where the effect of breed was controlled. Therefore, it is unlikely that the results were coincidental, i.e., due to allergic owners tending to select allergy-prone dog breeds.

**Table 4:** Association between allergy and current living environment in owners. Adult and child owners were treated separately, using logistic regression. Only children whose average ages were over three years were considered. When current living environment becomes more urban, odds to become allergic are higher. Odds Ratio, OR = exp(estimate).

Owners	Subset	*n*_*allergic*_	*n*_*healthy*_	OR	*P*
Adults	*Full data*	2654	3068	1.10	0.001
	*Allergy-tolerant breeds*	875	1003	1.10	0.004
	*Common breeds*	367	365	1.06	0.45
Children	*Full data*	431	742	1.18	0.011
	*Allergy-tolerant breeds*	269	507	1.16	0.056
	*Common breeds*	59	96	0.95	0.78

## Discussion

Our results indicate that rural (i.e. non-built) residential environments are associated with lower risk of owner-reported allergic symptoms in dogs. Moreover, several factors related to rural living, such as living in outdoor facilities and having regular contact with other animals (other pets and/or farm animals), are related to lower risk of allergic symptoms (i.e. high allergy score) in dogs. We also find similar patterns for dog owners, even concurrently, suggesting that lifestyle and living environment are important for mammalian health in general. The relationship between the living environment and allergies is already established in humans^5,34^, but studies focusing on this relationship in dogs are rare. However, our findings are consistent with those from previous studies. In Sweden, living in a town and being born in autumn were associated with an increased risk of CAD^17^. In Switzerland, living in rural environment, living in a household with other animals and walking regularly in a forest were associated with lower risk of CAD^21^. Our study adds further affirmation to the Swedish study, which was based on insurance company information^17^, with well-described environmental quality and exposure, and to the Swiss study by using a larger dataset^21^.

As noted above, breed is undeniably a very important factor in canine allergies^17,35–40^. Different dog breeds are not equally common in rural and urban environments, which could contribute to our result and previous findings. Therefore, we tried to control for the effect of breed by creating a subset including only common breeds, which were as prevalent in rural and urban environments, by excluding breeds with high predisposition to allergies, and other subset, from which we excluded all breeds previously found to be prone to develop allergies. We found a similar pattern in the relationship between the residential environment and allergic symptoms both in full data and in breed-controlled subsets. Therefore, we are confident that the living environment has a role in allergies in dogs, even though at individual level the predisposing effect of some breeds must be noticed.

It has been recently suggested that factors predisposing humans to allergies can be important also in dogs^41^. Here, we found family size and animal contacts to be such factors in canine allergies. Dogs were less allergic if they were living in families with more than two children. As pet dogs have previously been shown to be allergy protective^42–44^, our findings suggest that the protective effect might be bi-directional. Additionally, living in a farm and having regular contact with other animals (other pets and/or farm animals) was associated with healthier dogs, which has also been previously reported for humans^45^.These lifestyle features make it rather difficult to interpret the effect of residential land-use, as lifestyle and environment are often related. Moreover, their effects on health might be interactive as certain lifestyle factors may further increase contact with the surrounding environment.

Accumulating evidence suggest that early life is especially important regarding the development of allergic diseases in humans. For example, the nature-oriented lifestyle early in life seems to protect against allergic diseases and sensitization in children^46^. In dogs, the endotoxin exposure has been shown to inversely associate with CAD^47^. Moreover, prenatal exposure to farming environments tends to be related to future health in humans^5,48^. Still, in our data, the land use in the birth environment (i.e., in breeder’s home) was not associated with allergic symptoms in dogs. This disparity between our study and earlier human studies is likely to be explained by withdrawal age. Dogs are usually withdrawn at the age of seven to eight weeks, which means puppies are still developing in the environment of their new owners. Consequently, it may be that the early change from birth environment to current environment masks the effect of early life, as the current environment will also make an important contribution to the developing immune system. For example, those dogs, which had regularly had contact with other animals after withdrawal from their mother up to 6 months of age, were less allergic. In contrast, dogs that spent this time mostly in cities were more allergic.

Recently, the focus on allergy research has been on the role of microbiota, the diverse microbial community of the host. Increasing evidence suggests that changes in the composition of this community are related to several non-communicable diseases in humans^3^. In dogs, the skin microbiota of individuals suffering from allergies has been reported to differ from healthy individuals^49^. Also, the composition of skin microbiota has recently been shown to differ between urban and rural people^11,50^. The mechanisms of the interaction between microbiota and immune cells are being unraveled^51^ and there is increasing confidence about the causal role of microbiota in host health^3^. We suggest, in accordance with other studies^17,21^, that the higher allergy prevalence in dogs and their owners in urban environments is related to their limited contact with immune-modulating, environmental microbes. Moreover, not just the type of the environment, but several factors increasing individual’s contact with the surrounding environment are central in defining the amount and type of microbes dogs and humans are exposed to^5^. This suggests that negative associations between the exposure to rural environments and inflammatory disorders might be a general phenomenon in mammals, supporting the suggested beneficial role of microbes found from green environments for immune tolerance^9^.

If the origin of allergies in humans and dogs are causally related, one would expect similar immunological base of allergies in both species. However, in canine allergies neither the immunological mechanisms nor outcomes are as well-described as in humans. For example, the role of immunoglobulin E, a classic indicator of allergies in humans, is widely debated in allergies in dogs^52,53^. However, also in humans, there is more consensus on airborne allergens causing an IgE reaction rather than food allergens. On the other hand, dissimilar immunological processes producing similar non-communicable diseases may still have similar causal factors. In conclusion, further understanding of the mechanistic differences and similarities in mammalian allergies is needed.

Dogs are considered to be a good animal models for understanding human diseases^18,22,54,55^. Yet, laboratory mice and rats are often used to study common human diseases^56^. However, non-communicable diseases are usually multifactorial and complex, and thus simplified laboratory circumstances can be unable to reveal the whole image of these diseases. Moreover, many of these diseases do not occur spontaneously in laboratory mice and rats. Canine allergies mimic more closely the situation in humans than do rodent counterparts. Importantly, as the lifestyle and environmental factors have been suggested as key factors in the development of non-communicable diseases, dogs share the living environment and lifestyle with their owner. Therefore, dogs serve as a model with naturally occurring diseases, but still with a much simpler and shorter lifespan than humans.

The strength of survey studies is the access to a large number of individuals in geographically diverse areas with limited costs. However, survey-based studies have also common limitations. The survey may select for people who are already interested in the study question. In our case, the invitation letter may have selected those dog owners who had allergic dogs as in the invitation letter the focus of the study was mentioned. Importantly, in this study the dogs’ health is defined by their owners, not by veterinarians. Consequently, the estimation of allergic symptoms may differ between owners. For example, dogs living in outdoor facilities can be less carefully followed than indoor dogs, and hence their symptoms may not be noticed as easily.

Both in dogs and humans, the diet is likely to be an important factor in the development of allergies^57–59^. However, this dataset gives us only a coarse estimate of the current diet of the dogs, but we saw that allergic dogs eat more raw and veterinary-prescribed dog foods formulated for allergic dogs (*Full data: Χ*^2^ = 253.715, df = 9, *P* = 1.64e^−49^, *Allergy-tolerant: Χ*^2^ = 126.208, df = 9, *P* = 7.13e^−23^, *Common breeds: Χ*^2^ = 19.731, df = 9, *P* = 0.011). However, we doubt that neither of these food types are causal for canine allergies. Rather, allergic dogs tend to have a special diet in order to ease symptoms as there is anecdotal evidence that this will help to ameliorate the symptoms. The effect of diet on canine allergies needs to be further studied with interventions.

Human inflammatory disorders are increasing in Western societies^60,61^. In dogs, such a long-term data is scarce, but Nødtvedt *et al*. reported (2006) a slight increase in the incidence of atopic dermatitis^17^. Here, we have shown with a relatively large dataset, applying a breed-controlled analyses, that the allergic symptoms in dogs are inversely related to the proportion of rural space in their living environment. Several other factors related to nature-oriented or rural lifestyle also associate to allergic symptoms in dogs. Curiously, dogs and owners tend to share their health status, indicating mutual causal factors in the mammalian allergies. This study adds to the constantly increasing knowledge about the necessity of rural environment for individual health. Based on this knowledge, we recommend implementing several actions, such as planning greener cities, to increase our and our pets’ contact with green habitats.

## Materials and Methods

### Data collection

We conducted an owner-completed survey among Finnish dog owners (Supplementary form S1). Information on allergic history and environmental factors was collected using a 95-question survey focused on six aspects of dog’s life: basics, background, allergic signs, feeding, outdoor activities, and surroundings (Supplementary form S1). We also inquired the allergic symptoms of dog owners in the survey. The main method in sample collection was an email invitation to answer the questionnaire, which was sent to people who had provided information on their dog to the Canine Genetics research group of the University of Helsinki (www.koirangeenit.fi/english). In total, 17 262 emails were sent. This data was complemented by providing an open possibility for anyone interested to answer, as the survey was freely available on the website. The survey was also promoted by several breed organizations. It was open for about a year and a half during 2015 and 2016. In total, 5867 answers were collected (participation rate ~34%). To see whether the sample was representative or not, we tested how well our data resembled the registration information in Finland (Supplementary Table S1). The analysis indicated that the sample matched well to the actual breed frequencies (Wilcoxon signed rank test Z = -0.906, *P* = 0.337), suggesting that our data had a reasonable match with the actual dog population in Finland.

### Data editing

Identification data (names, addresses) was collected and prior to analysis any kind of identification information was removed. Some answers (*n* = 145) were removed from the data because of incomplete answers or because of multiple answers for the same dog. The final data set had 5722 dogs from 258 different breeds, 3059 of which were female (53%). The ages of the dogs varied from 3 months to 18 years (mean 6.1 years, SD 3.0 years). Many breeds (*n* = 28) were represented by only one individual, but 30 breeds had more than 50 individuals in the data (overall, the mean and SD of number of dogs per breed was 22.2 and 33.8, respectively). The total list of breeds and the number of individuals per breed in the data are provided in Supplementary Table S1.

### Definition of allergy score (allergic symptoms) in dogs and allergy in their owners

Due to the great variation in allergic symptoms in dogs^62,63^, no single question was considered a reliable measurement of canine allergy in this data. A large set of questions regarding allergic symptoms and their treatments (questions 5.1–6.11 in Supplementary Form S1) were used in defining an allergy score. This variable was calculated by simplifying survey information regarding symptoms and treatments to one continuous variable, using exploratory factor analysis (Promax rotation with Kaizer normalization was used since it permits correlation among factors). The first axis from the factor analysis was taken as the *allergy score (*the loadings of different questions are given the Supplementary Material Table S2). The resulting, continuous variable *allergy score* (with range from −1.07 to 4.31, mean 0.00, and SD 0.96) indicates the severity of allergic symptoms in individuals. Higher allergy scores correlate with more prominent allergic symptoms.

The continuous allergy score was analysed against the environmental land-use (see below) as well as against other environmental factors. The allergy score was also partitioned into three groups: healthy, possibly allergic, and allergic. We used previous literature as a base for defining the realistic prevalence of allergic dogs^32,62,64^. We determined the 10% of dogs with highest allergy score as “allergic” (allergy score ≥ 1.43) and the 75% with lowest allergy score as “healthy” (allergy score ≤ 0.39); dogs that fell between these two groups were considered “possibly allergic”. Categorical variable enables some analyses (for example, logistic regression) as we can focus on extreme groups: healthy (i.e. low allergy score -group) and allergic (i.e. high allergy score -group).

Owners were asked whether they, or other members in the family, have diagnosed allergic symptoms (questions 6.12–6.14 in Supplementary Form S1). The allergies in adult and child members of the family were considered separately. A person was defined allergic if he/she had either diagnosed allergic rhinitis, atopic eczema or asthma (categorical yes/no -variable). In case of children, we only analyzed those families which had children older than 3 years on average, since in younger children the allergic outcomes are sometimes vague.

### Land-use quantification

We quantified the environmental land-use around both the birth home and the current home of a dog. Geographical coordinates for each home were derived using addresses provided by dog owners. The coverage of seven land-use types (coastal wetlands; industrial, commercial and transport units; marine waters, open spaces with little or no vegetation; artificial and non-agricultural areas; mine, dump and construction sites; and urban fabric) were calculated within a three-kilometer buffer around the homes, using the publicly available land-use database CORINE2012, with 25 m resolution in Finland. This information was further simplified into one continuous variable (through principal component analysis with Varimax rotation and Kaizer normalization). This essentially reduced the land-use information to a rural–urban gradient (Supplementary Table 3). Higher values along this axis correlate with a larger proportion of built environment.

To form a categorical rural–urban factor we could not use the national definition of rural and urban environments. This is because the definition of city in Finland is very vague and would have been confounding as our study is interested in the dogs’ possibility to have close nature encounters. Instead, we used the proportion of urban fabric (ranging between 0–60%) to split the data in half: rural areas with less than 20% urban fabric and urban areas with more than 20% urban fabric (*n* = 2945 and 2777, respectively).

### Controlling for breed effects

Breed is a potential confounding factor in the analyses, as there are differences in the prevalence of inherited CAD disorders between breeds^35^. Predisposition of breeds may vary by study but some breeds are frequently mentioned, such as West Highland White Terrier, Boxer, German Shepherd Dog, Bull Terrier, French Bulldog, Labrador Retriever, and Golden Retriever^17,35–40^. Based on literature search, we created a list of allergy-prone breeds (Supplementary Table S4).

In attempt to control for breed effects we created two subsets. Into the first subset, we selected only the five most common breeds, which were not mentioned in previous studies as allergy-prone, being represented by over 100 individuals in our data (*Common breeds*, including: Border Collie, Finnish Lapphund, Lagotto Romagnolo, Shetland Sheepdog and mixed-breed dogs, *n* = 732, Supplementary Table S1). For the second subset, we excluded the breeds mentioned in the allergy-prone list (*Allergy-tolerant breeds*, 211 breeds, *n* = 3844, Supplementary Table S1). This approach enabled us to test whether results obtained for the full data were driven by either allergy-prone or common breeds, retaining as much original data as possible. In total, we had 572 allergic dogs in the *full data*, 39 in *Common breeds* subset, and 284 in *Allergy-tolerant breeds* subset.

### Statistical analysis

Due to many behavioral and lifestyle-associated variables being strongly correlated with each other, we used factor analysis to extract three independent variables: agricultural lifestyle, outdoor exposure, and outdoor activities. *Agricultural lifestyle* reflected current agricultural activity and contact with farm animals, *outdoor exposure* reflected drinking from waterways, licking urine, and eating dirt, grass and faeces while on a walk, and *outdoor activities* reflected the freedom of movement and the type of the exercise environments (Supplementary Table S5).

Logistic regression was used to analyse whether allergy prevalence of the dogs and humans (i.e. the relative proportions of allergic and healthy individuals) was associated with the living environment. Mann-Whitney or Kruskal-Wallis tests were used to assess the effect of several explanatory factors, such as animal contacts and living conditions (full list in Table 2) on the allergy score in dogs. We further excluded breeds with less than 50 individuals from allergy-tolerant subset in order to create new subset that enables mixed modelling. With this subset, we examined the effect of various factors on dog allergy score simultaneously with a linear mixed model, controlling for residual dependence within breed and sex (random factors), using the *lmer* function in package *lme4* in R^65^. The association between the presence of allergy in owners and dogs was tested with *χ*^2^ -tests. Statistical analyses were done using the IBM SPSS statistics software version 24.0 and R version 3.3.2^66^.

## Electronic supplementary material

Supplementary information
