# Supplementary material for: Urban environment predisposes dogs and their owners to allergic symptoms
Source: Sci Rep. 2018 Jan 25;8:1585. doi: 10.1038/s41598-018-19953-3 (PMC5785484; doi:10.1038/s41598-018-19953-3)
Supplement: Supplementary file 1 — Supplementary information [file 41598_2018_19953_MOESM1_ESM.pdf]

SUPPLEMENTARY MATERIAL

Urban environment predisposes dogs and their owners to allergic symptoms

Emma Hakanen[1], Jenni Lehtimäki[1*], Elina Salmela[1,2], Katriina Tiira[2], Johanna Anturaniemi[3], Anna Hielm-Björkman[3], Lasse Ruokolainen[1] and Hannes Lohi[2]

1 Department of Biosciences, University of Helsinki, PL 65 FI-00014 Helsinki, Finland.
2 Research Programs Unit, Molecular Neurology, and Department of Veterinary Biosciences, University of Helsinki, Helsinki, Finland; The Folkhälsan Institute of Genetics, Helsinki, Finland
3 Department of Equine and Small Animal Medicine, University of Helsinki, PL 57, FI-00014 Helsinki, Finland

*To whom correspondence should be addressed: Jenni Lehtimäki, PL 65, FI-00014 Helsinki, Finland, +358504480047, jenni.lehtimaki@helsinki.fi

Table S1. This table shows all the breeds in total data, the number of individuals per breed, and the breeds' prevalence in this study (ns and %s, respectively). The data in this study is compared to the statistics about dog breeds in Finland: the number of individuals in each breed in Finland is provided (np) as well as the prevalence of each breed in Finnish population (%p). Finally, the ratio of breeds' prevalence in our data and their prevalence in Finland is provided (%-ratio). 'No data' means that the breed is not officially registered by the Finnish Kennel Club. Breeds in the *Allergy-tolerant* and *Common breeds* subsets are marked with *italicized* and **bolded** text, respectively.

Breed	n_S	$\%_S$	n_P	$\%_P$	%-ratio
Affenpinscher	6	0.0010	363	0.0008	1.39
Afghan Hound	34	0.0059	610	0.0013	4.68
Airedale Terrier	30	0.0052	747	0.0016	3.37
Akita	24	0.0042	524	0.0011	3.85
Alaskan Malamute	10	0.0017	1523	0.0032	0.55
Alpine Dachsbracke	2	0.0003	20	0.0000	8.40
American Akita	13	0.0023	1381	0.0029	0.79
American bulldog	2	0.0003	no data	no data	no data
American Cocker Spaniel	15	0.0026	1292	0.0027	0.98
American Hairless Terrier	10	0.0017	no data	no data	no data
American Staffordshire Terrier	36	0.0063	1823	0.0038	1.66
American Water Spaniel	1	0.0002	46	0.0001	1.83
Appenzeller Sennenhund	10	0.0017	260	0.0005	3.23
Australian Cattle Dog	7	0.0012	313	0.0007	1.88
Australian Kelpie	36	0.0063	784	0.0016	3.86
Australian Shepherd	59	0.0103	2650	0.0055	1.87
Australian Silky Terrier	14	0.0024	1098	0.0023	1.07
Australian Terrier	31	0.0054	2375	0.0049	1.10
Barbet	22	0.0038	381	0.0008	4.85
Basenji	19	0.0033	730	0.0015	2.19
Basset Artésien Normand	1	0.0002	37	0.0001	2.27
Basset Fauve de Bretagne	16	0.0028	520	0.0011	2.59
Basset Hound	5	0.0009	636	0.0013	0.66
Bavarian Mountain Hound	1	0.0002	46	0.0001	1.83
Beagle	40	0.0070	5833	0.0121	0.58
Bearded Collie	31	0.0054	653	0.0014	3.99
Beauceron	12	0.0021	743	0.0015	1.36
Bedlington Terrier	3	0.0005	281	0.0006	0.90
Belgian Shepherd Dog Groenendael	47	0.0082	1326	0.0028	2.98
Belgian Shepherd Dog Laekenois	7	0.0012	59	0.0001	9.97
Belgian Shepherd Dog Malinois	45	0.0079	1739	0.0036	2.17
Belgian Shepherd Dog Tervueren	51	0.0089	1554	0.0032	2.76
Bernese Mountain Dog	81	0.0142	4696	0.0098	1.45
Bichon Frisé	46	0.0080	6308	0.0131	0.61
Black Russian Terrier	20	0.0035	504	0.0010	3.33
Bloodhound	1	0.0002	71	0.0001	1.18
Blue Picardy Spaniel	2	0.0003	9	0.0000	18.67
Bohemian Shepherd	3	0.0005	98	0.0002	2.57
Bolognese	2	0.0003	869	0.0018	0.19
Bolonka	1	0.0002	58	0.0001	1.45
Border Collie	104	0.0182	4190	0.0087	2.09
Border Terrier	27	0.0047	2584	0.0054	0.88
Borzoi	31	0.0054	864	0.0018	3.01
Boston Terrier	22	0.0038	1650	0.0034	1.12
Bouvier des Ardennes	1	0.0002	1	0.0000	84.03

Bouvier des Flandres	12	0.0021	474	0.0010	2.13
Boxer	73	0.0128	2753	0.0057	2.23
Bracco Italiano	6	0.0010	185	0.0004	2.73
Braque du Bourbonnais	2	0.0003	64	0.0001	2.63
Brazilian Terrier	13	0.0023	1308	0.0027	0.84
Briard	7	0.0012	421	0.0009	1.40
Brittany	8	0.0014	1076	0.0022	0.62
Broholmer	7	0.0012	115	0.0002	5.11
Bull Terrier	28	0.0049	621	0.0013	3.79
Bulldog	18	0.0031	2198	0.0046	0.69
Bullmastiff	13	0.0023	1328	0.0028	0.82
Cairn Terrier	51	0.0089	4567	0.0095	0.94
Canaan Dog	1	0.0002	38	0.0001	2.21
Cane Corso	5	0.0009	479	0.0010	0.88
Catalonian Sheepdog	5	0.0009	204	0.0004	2.06
Caucasian Shepherd Dog	3	0.0005	644	0.0013	0.39
Cavalier King Charles Spaniel	33	0.0058	7531	0.0157	0.37
Central Asia Shepherd Dog	1	0.0002	300	0.0006	0.28
Cesky Terrier	4	0.0007	108	0.0002	3.11
Chihuahua	21	0.0037	13548	0.0282	0.13
Chinese Crested Dog	46	0.0080	4722	0.0098	0.82
Chow Chow	20	0.0035	1238	0.0026	1.36
Chukotka sleddog	1	0.0002	no data	no data	no data
Cirneco dell'Etna	13	0.0023	279	0.0006	3.92
Clumber Spaniel	1	0.0002	183	0.0004	0.46
Collie Rough	66	0.0115	6513	0.0135	0.85
Collie Smooth	40	0.0070	2050	0.0043	1.64
Continental Toy Spaniel	13	0.0023	3788	0.0079	0.29
Coton de Tulear	58	0.0101	5451	0.0113	0.89
Croatian Shepherd Dog	2	0.0003	43	0.0001	3.91
Curly Coated Retriever	24	0.0042	399	0.0008	5.05
Czechoslovakian Wolfdog	2	0.0003	194	0.0004	0.87
Dachshund Long-Haired	17	0.0030	1948	0.0041	0.73
Dachshund Short-Haired	11	0.0019	2888	0.0060	0.32
Dachshund Wire-Haired	32	0.0056	7276	0.0151	0.37
Dalmatian	21	0.0037	1850	0.0038	0.95
Dandie Dinmont Terrier	9	0.0016	339	0.0007	2.23
Danish-Swedish Farmdog	26	0.0045	998	0.0021	2.19
Deerhound	2	0.0003	272	0.0006	0.62
Deutsch Drahthaar	21	0.0037	2865	0.0060	0.62
Dobermann	23	0.0040	2113	0.0044	0.91
Dogo Argentino	5	0.0009	457	0.0010	0.92
Dogue de Bordeaux	3	0.0005	404	0.0008	0.62
Do-Khyi	5	0.0009	865	0.0018	0.49
Drentsche Partrigde Dog	1	0.0002	3	0.0000	28.01
Drever	12	0.0021	2300	0.0048	0.44
Dutch Schapendoes	9	0.0016	905	0.0019	0.84
Dutch Shepherd Dog	23	0.0040	870	0.0018	2.22
East Siberian Laika	7	0.0012	3104	0.0065	0.19
English Cocker Spaniel	29	0.0051	6842	0.0142	0.36
English Pointer	3	0.0005	325	0.0007	0.78
English Setter	7	0.0012	861	0.0018	0.68
English Springer Spaniel	30	0.0052	3166	0.0066	0.80
English Toy Terrier	7	0.0012	482	0.0010	1.22

Entlebuch Cattle Dog	2	0.0003	156	0.0003	1.08
Estonian Hound	2	0.0003	379	0.0008	0.44
Estrela Mountain Dog	9	0.0016	418	0.0009	1.81
Eurasian	5	0.0009	580	0.0012	0.72
Field Spaniel	5	0.0009	464	0.0010	0.91
Finnish Hound	39	0.0068	17909	0.0372	0.18
Finnish Lapphund	254	0.0444	11929	0.0248	1.79
Finnish Spitz	49	0.0086	7358	0.0153	0.56
Flat Coated Retriever	70	0.0122	2642	0.0055	2.23
Fox Terrier Smooth	9	0.0016	1636	0.0034	0.46
Fox Terrier Wire	15	0.0026	1200	0.0025	1.05
French Bulldog	45	0.0079	4544	0.0095	0.83
German Hunting Terrier	7	0.0012	1716	0.0036	0.34
German Pinscher	30	0.0052	867	0.0018	2.91
German Shepherd Dog	310	0.0542	17394	0.0362	1.50
German Short-Haired Pointing Dog	26	0.0045	2425	0.0050	0.90
Giant Schnauzer	28	0.0049	1522	0.0032	1.55
Golden Retriever	108	0.0189	13953	0.0290	0.65
Gordon Setter	7	0.0012	823	0.0017	0.71
Grand Basset Griffon Vendeen	3	0.0005	222	0.0005	1.14
Great Dane	52	0.0091	3169	0.0066	1.38
Great Swiss Mountain Dog	1	0.0002	95	0.0002	0.88
Greyhound	4	0.0007	405	0.0008	0.83
Griffon Belge	2	0.0003	259	0.0005	0.65
Griffon Bruxellois	2	0.0003	467	0.0010	0.36
Havanese	34	0.0059	5193	0.0108	0.55
Hellenic Hound	1	0.0002	no data	no data	no data
Hovawart	17	0.0030	2052	0.0043	0.70
Hungarian Greyhound	2	0.0003	85	0.0002	1.98
Ibizan Podenco	6	0.0010	102	0.0002	4.94
Icelandic Sheepdog	16	0.0028	596	0.0012	2.26
Irish Glen of Imaal Terrier	6	0.0010	512	0.0011	0.98
Irish Red and White Setter	2	0.0003	131	0.0003	1.28
Irish Red Setter	26	0.0045	2476	0.0051	0.88
Irish Soft Coated Wheaten Terrier	59	0.0103	1445	0.0030	3.43
Irish Terrier	4	0.0007	402	0.0008	0.84
Irish Water Spaniel	5	0.0009	152	0.0003	2.76
Irish Wolfhound	22	0.0038	1465	0.0030	1.26
Italian Greyhound	26	0.0045	807	0.0017	2.71
Italian Short-Haired Segugio	1	0.0002	16	0.0000	5.25
Jack Russel Terrier	72	0.0126	8684	0.0181	0.70
Japanese Spitz	12	0.0021	1735	0.0036	0.58
Jämthund	21	0.0037	12780	0.0266	0.14
Kai	2	0.0003	16	0.0000	10.50
Karelian Bear Dog	41	0.0072	7120	0.0148	0.48
Keeshond	17	0.0030	1136	0.0024	1.26
Kerry Blue Terrier	7	0.0012	403	0.0008	1.46
King Charles Spaniel	4	0.0007	230	0.0005	1.46
Kleiner Münsterländer	6	0.0010	482	0.0010	1.05
Kooikerhondje	40	0.0070	833	0.0017	4.03
Koolie	1	0.0002	no data	no data	no data
Kromfohrländer	20	0.0035	369	0.0008	4.55
Labrador Retriever	126	0.0220	17913	0.0373	0.59
Lagotto Romagnolo	121	0.0211	3122	0.0065	3.26

Lakeland Terrier	2	0.0003	135	0.0003	1.24
Lancashire Heeler	28	0.0049	958	0.0020	2.46
Landseer	9	0.0016	1091	0.0023	0.69
Lapponian Herder	95	0.0166	2490	0.0052	3.21
Large Munsterlander	5	0.0009	80	0.0002	5.25
Leonberger	39	0.0068	2582	0.0054	1.27
Lhasa Apso	1	0.0002	802	0.0017	0.10
Little Lion Dog	5	0.0009	489	0.0010	0.86
Maltese	2	0.0003	617	0.0013	0.27
Manchester Terrier	9	0.0016	361	0.0008	2.09
Mastiff	5	0.0009	294	0.0006	1.43
Medium Poodle	32	0.0056	3018	0.0063	0.89
Medium Size Spitz	13	0.0023	4993	0.0104	0.22
Miniature Bull Terrier	13	0.0023	340	0.0007	3.21
Miniature Dachshund Long-Haired	16	0.0028	2220	0.0046	0.61
Miniature Dachshund Short-Haired	5	0.0009	1291	0.0027	0.33
Miniature Dachshund Wire-Haired	6	0.0010	1617	0.0034	0.31
Miniature Pinscher	53	0.0093	5451	0.0113	0.82
Miniature Poodle	26	0.0045	4595	0.0096	0.48
Miniature Schnauzer	95	0.0166	9778	0.0203	0.82
Miniature Spitz	19	0.0033	1911	0.0040	0.84
mixed-breed	111	0.0194	no data	no data	no data
Mudi	14	0.0024	357	0.0007	3.30
Newfoundland	36	0.0063	2152	0.0045	1.41
Norbottenspitz	17	0.0030	2793	0.0058	0.51
Norfolk Terrier	10	0.0017	972	0.0020	0.86
Norwegian Buhund	7	0.0012	77	0.0002	7.64
Norwegian Elkhound Grey	29	0.0051	10213	0.0212	0.24
Norwegian Lundehund	2	0.0003	254	0.0005	0.66
Norwich Terrier	12	0.0021	1257	0.0026	0.80
Nova Scotia Duck Tolling Retriever	72	0.0126	3610	0.0075	1.68
Old English Sheepdog	14	0.0024	386	0.0008	3.05
Otterhound	1	0.0002	24	0.0000	3.50
Parson Russel Terrier	35	0.0061	3798	0.0079	0.77
Peruvian Hairless Dog	4	0.0007	412	0.0009	0.82
Petit Basset Griffon Vendeen	10	0.0017	526	0.0011	1.60
Petit Brabaḉon	8	0.0014	561	0.0012	1.20
Pharaoh Hound	9	0.0016	251	0.0005	3.01
Picardy Sheepdog	15	0.0026	68	0.0001	18.54
Picardy Spaniel	1	0.0002	17	0.0000	4.94
Polish Greyhound	3	0.0005	43	0.0001	5.86
Polish Lowland Sheepdog	1	0.0002	251	0.0005	0.33
Pomeranian	8	0.0014	1911	0.0040	0.35
Pont-Audemer Spaniel	2	0.0003	34	0.0001	4.94
Portuguese Podengo	1	0.0002	45	0.0001	1.87
Portuguese Sheepdog	3	0.0005	534	0.0011	0.47
Portuguese Water Dog	17	0.0030	1281	0.0027	1.12
Pražský krysařík	3	0.0005	188	0.0004	1.34
Pug	30	0.0052	3447	0.0072	0.73
Puli	2	0.0003	321	0.0007	0.52
Pumi	21	0.0037	764	0.0016	2.31
Pyrenean Mastiff	11	0.0019	1136	0.0024	0.81
Pyrenean Mountain Dog	7	0.0012	602	0.0013	0.98
Pyrenean Sheepdog Long Haired	7	0.0012	284	0.0006	2.07

Pyrenean Sheepdog Smooth-Faced	8	0.0014	105	0.0002	6.40
Rabbit Dachshund Long-Haired	5	0.0009	356	0.0007	1.18
Rhodesian Ridgeback	43	0.0075	807	0.0017	4.48
Rottweiler	74	0.0129	4721	0.0098	1.32
Russian Hound	1	0.0002	864	0.0018	0.10
Russian Toy	5	0.0009	959	0.0020	0.44
Russian-European Laika	3	0.0005	355	0.0007	0.71
Saarloos Wolfhond	2	0.0003	169	0.0004	0.99
Saluki	59	0.0103	944	0.0020	5.25
Samoyed	46	0.0080	2263	0.0047	1.71
Schipperke	31	0.0054	1316	0.0027	1.98
Schnauzer	27	0.0047	1411	0.0029	1.61
Scottish Terrier	6	0.0010	611	0.0013	0.83
Shetland Sheepdog	142	0.0248	9467	0.0197	1.26
Shiba	14	0.0024	633	0.0013	1.86
Shih Tzu	3	0.0005	958	0.0020	0.26
Siberian Husky	19	0.0033	5599	0.0116	0.29
Skye Terrier	4	0.0007	352	0.0007	0.95
Sloughi	2	0.0003	67	0.0001	2.51
Slovakian Chuvach	3	0.0005	259	0.0005	0.97
Small Portuguese Podengo	13	0.0023	971	0.0020	1.12
Southh Russian Shepherd Dog	1	0.0002	122	0.0003	0.69
Spanish Greyhound	2	0.0003	45	0.0001	3.73
Spanish Mastiff	1	0.0002	134	0.0003	0.63
Spanish Water Dog	59	0.0103	3962	0.0082	1.25
Spinone Italiano	3	0.0005	129	0.0003	1.95
St. Bernard	19	0.0033	1163	0.0024	1.37
Stabijhoun	9	0.0016	362	0.0008	2.09
Staffordshire Bull Terrier	73	0.0128	3299	0.0069	1.86
Standard Poodle	16	0.0028	1098	0.0023	1.22
Swedish Elkhound White	1	0.0002	410	0.0009	0.20
Swedish Lapphund	1	0.0002	57	0.0001	1.47
Swedish Vallhund	28	0.0049	1145	0.0024	2.05
Thai Ridgeback	1	0.0002	63	0.0001	1.33
Tibetan Spaniel	25	0.0044	7990	0.0166	0.26
Tibetan Terrier	10	0.0017	1330	0.0028	0.63
Toy Fox Terrier	1	0.0002	22	0.0000	3.82
Toy Poodle	4	0.0007	1853	0.0039	0.18
Weimaraner	3	0.0005	363	0.0008	0.69
Welsh Corgi Cardigan	12	0.0021	2047	0.0043	0.49
Welsh Corgi Pembroke	16	0.0028	2563	0.0053	0.52
Welsh Springer Spaniel	46	0.0080	2335	0.0049	1.66
Welsh Terrier	5	0.0009	191	0.0004	2.20
West Highland White Terrier	51	0.0089	4920	0.0102	0.87
West Siberian Laika	2	0.0003	701	0.0015	0.24
Whippet	63	0.0110	2443	0.0051	2.17
White Swiss Shepherd Dog	66	0.0115	2889	0.0060	1.92
Vizsla	6	0.0010	323	0.0007	1.56
Volpino Italiano	2	0.0003	367	0.0008	0.46
Xoloitzcuintle	1	0.0002	78	0.0002	1.08
Yorkshire Terrier	2	0.0003	2531	0.0053	0.07

Table S2. The variables (allergic symptoms) used in the definition of allergy score. Second column shows the contribution of each variable for the first extracted factor "allergy score" (allergic symptoms), which was selected for further analysis.

Allergic symptoms	Contribution
Allergic skin symptoms	0.167
Atopic dermatitis symptoms	0.106
Other allergic or atopic symptoms	0.072
Demodex symptoms	-0.024
Food allergy symptoms	0.096
Pruritus symptoms	0.138
Intensity of pruritus symptoms	0.737
Pruritus symptoms: corner of the mouth	0.487
Pruritus symptoms: inner skin of the ear pinna	0.474
Pruritus symptoms: outer margins of the ear	0.299
Pruritus symptoms: dorsal side of the snout	0.316
Pruritus symptoms: eyelid	0.320
Pruritus symptoms: neck/throat	0.446
Pruritus symptoms: armpit or axilla	0.528
Pruritus symptoms: interdigital or between toes	0.493
Pruritus symptoms: abdomen	0.608
Pruritus symptoms: groin or inguinal area	0.624
Pruritus symptoms: perineal area	0.474
Pruritus symptoms: flanks	0.426
Pruritus symptoms: back	0.425
Pruritus symptoms: tail	0.403
Pruritus symptoms: feet or pads	0.483
Pruritus symptoms: head	0.211
Pruritus symptoms: feet	0.322
Pruritus symptoms: elbow	0.491
Show first pruritus or skin symptoms	0.222
Scratch or rub snout	-0.057
Scratch, rub or lick corner of the mouth	0.033
Scratch or rub eyes	-0.078
Scratch or rub ears	0.013
Scratch, rub or lick feet	0.032
Scratch, rub, eat or lick groins or inguinal areas	0.080
Lick, bite or rub caudal back	-0.004
Scratch, rub, eat or lick all over	0.100
Show eczema or redness: not at all	-0.572
Show eczema or redness: around the snout	0.296
Show eczema or redness: at the corners of the mouth	0.438
Show eczema or redness: around the eyes	0.308
Show eczema or redness: in the ears	0.545
Show eczema or redness: between the toes	0.503
Show eczema or redness: under the feet	0.435
Show eczema or redness: on the abdomen	0.673
Show eczema or redness: in joint folds	0.665
Show eczema or redness: on other skin areas	0.358
Sneezing symptoms	-0.088
Cough symptoms	-0.042
Running eyes symptoms	-0.125
Diarrhea symptoms	-0.127
Gas/flatulance symptoms	-0.138
Constipation symptoms	-0.053
Vomiting symptoms	-0.130
Diagnosed: eye infections	0.251
Diagnosed: urticaria	0.367
Diagnosed: recurring ear infections	0.504
Diagnosed: recurring skin infections	0.669
Diagnosed: recurring "hot spots"	0.356
Diagnosed: not easily cured skin infections	0.532
Diagnosed: skin turning black	0.578
Diagnosed: alopecia or thinning hair coat	0.514
Diagnosed: skin thickening	0.293

Table S3. The contribution of different land-use types for the first axis "urban–rural gradient", which was used in further analyses. We measured the prevalence of different land-use types within 3 km distance from the current and birth homes.

Extracted factors	Current	Birth
Artificial areas and non-agricultural, vegetated areas	0.579	0.452
Coastal wetlands	-0.029	-0.004
Industrial, commercial, and transport units	0.914	0.913
Marine waters	0.254	0.003
Open spaces with little or no vegetation	0.063	0
Urban fabric	0.928	0.93
Mine, dump and construction sites	0	0.378

Table S4. Literature search about the allergy-prone breeds. Table shows the origin (country) of the study, breeds which were defined allergy-prone in the study, and the number of studies that defined each breed as allergy-prone (Total mentions). Breeds are organised by total mentions (N and % show absolute and relative prevalence in this study). Used studies are Tarpataki et al. 2006 (Hungary), Nødtvedt et al. 2006 (Sweden), Lund 2008 (Oregon), Picco et al. 2008 (Switzerland), Jaeger et al. 2010 (Australia, California_2, Germany) and Bellumori et al. 2013 (California_1).

Breed	Oregon (USA)	California_1 (USA)	California_2 (USA)	Sweden	Hungary	Switzerland	Germany	Australia	# of mentioned	N	%
West Highland White Terrier	x	x		x	x	x		x	6	51	0.35
Boxer	x			x	x	x		x	5	73	0.19
German Shepherd Dog				x	x		x	x	4	310	0.17
Bull Terrier				x		x		x	3	28	0.36
French bulldog					x	x	x		3	45	0.36
Labrador Retriever				x			x	x	3	126	0.13
Golden Retriever			x				x	x	3	108	0.12
American Staffordshire Terrier				x	x				2	36	0.33
Dalmatian				x	x				2	21	0.24
Rhodesian Ridgeback				x		x			2	43	0.19
Vizsla					x	x			2	6	0.17
Staffordshire Bull Terrier	x			x					2	73	0.15
Newfoundland				x	x				2	36	0.11
Jack Russel Terrier	x						x		2	72	0.08
Cairn Terrier	x	x							2	51	0.08
Bichon Frisé	x		x						2	46	0.02
American Bulldog	x								1	2	0.50
Puli					x				1	2	0.50
Bulldog	x								1	18	0.33
Tibetan Terrier		x							1	10	0.30
English Setter					x				1	7	0.29
Boston Terrier	x								1	22	0.27
Dobermann					x				1	23	0.22
Havanese	x								1	34	0.21

Poodle					x				1	49	0.18
Danish-Swedish Farmdog				x					1	26	0.15
Irish Soft Coated Wheaten Terrier				x					1	59	0.15
Welsh Springer Spaniel				x					1	46	0.15
Chow Chow					x				1	20	0.15
Pumi					x				1	21	0.14
Old English Sheepdog					x				1	14	0.14
Great Dane								x	1	52	0.08
Miniature Schnauzer	x								1	95	0.07
Fox Terrier Wire		x							1	15	0.07
Cavalier King Charles Spaniel								x	1	33	0.06
Dachshund					x				1	60	0.05
English Cocker Spaniel					x				1	29	0.03
Schnauzer	x								1	27	0.00
Australian Silky Terrier								x	1	14	0.00
Welsh Terrier				x					1	5	0.00
Shih Tzu	x								1	3	0.00
Maltese	x								1	2	0.00
Yorkshire Terrier	x								1	2	0.00
Lhasa Apso	x								1	1	0.00

Table S5. The factors forming the three independent environmental factors (outdoor exposure, outdoor activities, and agricultural lifestyle) used in the model of Table 3. The independent factors were extracted by factor analysis.

	Outdoor exposure	Outdoor activities	Agricultural lifestyle
Dirt eating outdoors	0.543		
Grass eating outdoors	0.524		
Feces eating and urine licking outdoors	0.517		
Drinking from waterways	0.410		
Freedomness of movement		0.745	
Environment outdoors		0.581	
Agriculture at current home			0.588
Contact with farm animals			0.500

Form S1. Translated questionnaire form used in study.

The residential environments and canine allergy-survey

In this survey, we study how living environment of a dog influence on canine allergies, which has increased recently.

Please, be honest with your answers: We want to study factors affecting dog health, not to evaluate anyone's dog keeping.

We hope that you answer this survey even though your dog is not having allergic disease.

All information is processed confidentiality and following the principles of law. Address information is used for definition of land use types around residential areas.

<u>Basic information of dog and owner</u>

2.1. First name of owner:

2.2. Last name of owner:

2.3. Street address:

2.4. Postal code:

2.5. Postal city:

2.6. Dog has moved with current owners

1. never
2. once
3. twice
4. thrice
5. more than three times

2.7. If dog have moved, write previous addresses:

2.8. Phone number:

2.9. Email:

2.10. The official name of dog:

2.11. The nick name of dog:

2.12. The breed of dog:

2.13. The register number of dog:

The birthday of dog:

2.14. Day:

2.15. Month:

2.16. Year:

2.17. If dog has died, write the date and cause of death:

2.18. The sex of dog:

1. Female
2. Male

2.19. Has dog been sterilized or castrated?

1. Yes
2. No

2.20. How large proportion of the dog's fur is white?

1. 0 %
2. about 25 %
3. about 50 %
4. about 75 %
5. 100 %

2.21. Have you given a blood sample of your dog for the Canine Genetics research group of the University of Helsinki?

1. Yes
2. No

Background of the dog

3.1. How many weeks old was the dog when arriving to current owners?

3.2. Where was your dog born and/or spent its first weeks?

1. I do not know
2. Dog is born in its current home
3. Dog is born in address mentioned below

3.3. The street address of birth home:

3.4. The postal code of birth home:

3.5. The postal city of birth home:

3.6. The dog was born and/or spent its first weeks

1. In city center
2. In municipality center
3. In Conurbation of urban area
4. In Conurbation of country-side
5. In sparsely populated area
6. I do not know

3.7. In what kind of building has your dog is born and/or spent its first weeks

1. In flat/apartment
2. In row house
3. In town house
4. In farm house

5. In outdoor facilities
6. I do not know

3.8. Was there agricultural activities in your dog's breeder house while dog was there?

1. I do not know
2. No
3. Yes, a part-time
4. Yes, a full-time

3.9. Did your dog's dam give a birth and take care of her puppies in dam's home or somewhere else?

1. I do not know
2. Dam gave birth somewhere else than its current home (dam is a co-owned dog)
3. Dam gave birth in its current home

3.10. In which age (in weeks) your dog was weaned from its dam?

3.11. If you know what kind of food the dam was eating while being pregnant and nursing, describe here:

3.12. If you know, what kind of food your dog was eating before weaning age, describe here:

3.13. Has your dog experienced things described below after withdrawal from its dam up to 4 months of age? Mark the most adequate option in all sections.

	I do not know	Never	Few times	Several times in a month	Several times in a week	Several times in a day
Dog met strange adult dogs						
Dog visited cities or other busy places						
Dog travelled by car						
Dog travelled by bus or train						
Dog met strange humans						
Dog met farm animals						

3.14. How much were your dog outdoors under two months age (before moving to you)?

1. I do not know
2. Not at all
3. Couple times in a month
4. Few times in a week
5. Once in a day

6. Many times in a day
7. Dog could move freely to outside

3.15. How much were your dog outdoors daily (in hours) when it was..

	I do not know	Not at all	0.5 hour	1 hour	2 hours	3 hours or more
3-4 months						
5-6 months						
7-12 months						

Dog practices

4.1. If your dog is a bitch, does it have puppies?

1. Yes
2. No

4.2. If your dog is a bitch and it have had puppies, mark here the litter details:

4.3. Is your dog a work dog?

1. My dog is not a work dog
2. Police dog
3. Border patrol dog
4. Guide dog
5. Drug dog
6. Service dog
7. Hearing dog
8. Mold detection dog
9. Other work dog (define below)

4.4. If you answered that your dog is other work dog, what kind of work does your dog do?

4.5. Do you have hobbies with your dog?

1. Yes
2. No

4.6. If you have hobbies with your dog, what kind of they are?

4.7. How much does your dog travel outside of your normal home conditions for example to dog shows, practices, competitions or work?

1. Not at all
2. 1-6 times in a year
3. 1-3 times in a month
4. 1-3 times in a week
5. Almost daily

4.8. What is the usual purpose of these travels?

Allergic symptoms and diseases of the dog, Part 1/2

5.1. Has your dog ever had any of the following diseases?

	No	Yes, according to breeder	Yes, according to vet	Yes, according to own judgement	Yes, according to somebody else (define to next section)	Who?
Allergic skin symptoms						
Atopic dermatitis						
Any other allergic or atopy related symptoms						
Demodicosis or Demodectic mange						
Food intolerance or food sensibility						

5.2. Has your dog ever suffered from scratching / pruritus?

1. Not at all
2. Little (once in a month - once in a week)
3. Moderately (once in a week - every second day)
4. Regularly (daily)

5.3. Estimate the severity of your dog's scratching when it is "on", using the scale below. Please use a number to evaluate the severity of the scratching (e.g. 22 or 87):

100	**Extremely intensive/almost constant scratching**<br>Nothing than physical barrier (e.g. collar) makes the dog stop the scratching/licking/biting
90	
80	**Intensive scratching/lengthened scratching periods**<br>Scratching can appear in nights (can be detected if dog is being monitored). Dog itches also when it is eating, playing or being active.
70	
60	**Moderate scratching/regularly repetitive scratching periods**<br>Scratching can appear in nights (can be detected if dog is being monitored). Dog does not itch when it is eating, playing or being active.
50	
40	**Mild scratching/slightly more frequent scratching periods**<br>Scratching does not appear in nights or when dogs is eating, playing or being active
30	
20	**Very mild scratching/only random scratching periods**<br>Dog itches only slightly more than normal
10	
0	**Normal dog**

	Dog does not itch more than normal, healthy dog

5.4. Pick those sections (using the picture below) that have sometimes itched in your dog:

A. Corner of the mouth
B. Inner skin of the ear pinna
C. Outer margins of the ear
D. Dorsal side of the snout
E. Eyelid
F. Neck / throat
G. Armpit or axilla
H. Interdigital or between toes
I. Abdomen
J. Groin or inguinal area
K. Perineal area
L. Flanks
M. Back
N. Tail
O. Feet, pads
P. Head
Q. Feet
R. Elbow

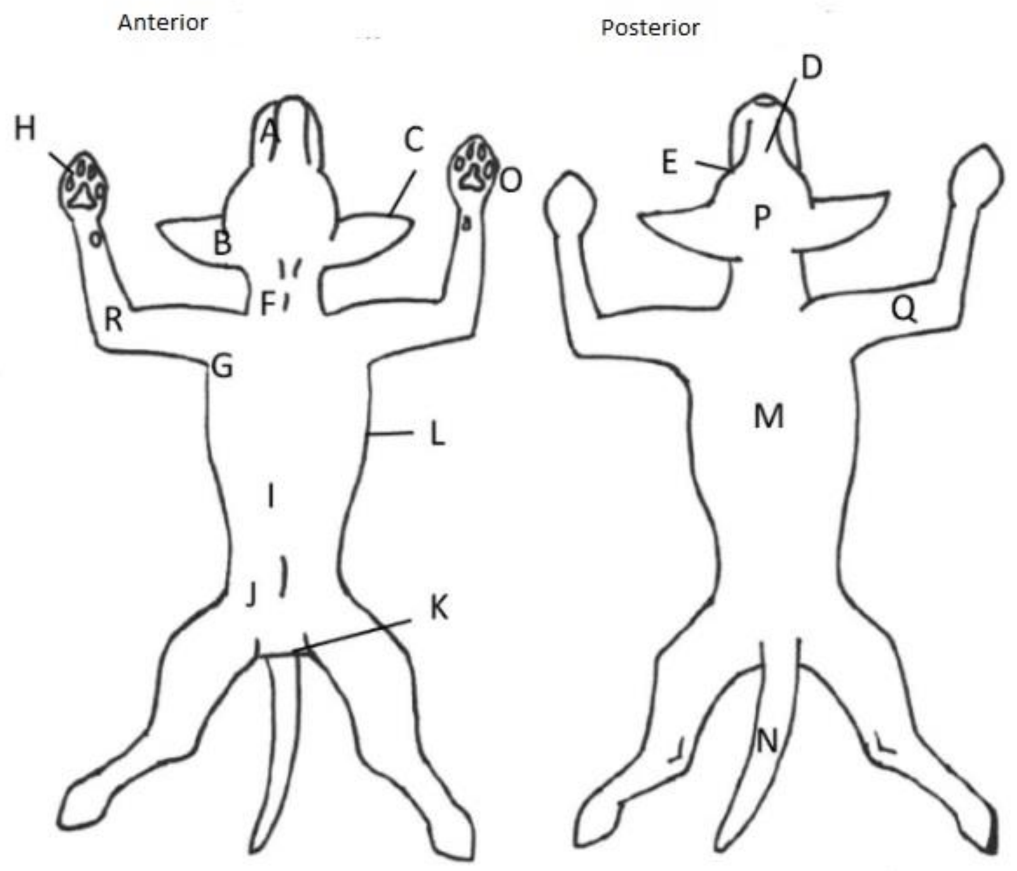

5.5. If your dog has both suffered from scratching and has had visual skin symptoms, which of them started first?

1. My dog had visual skin symptoms before scratching
2. My dog had scratching before visual skin symptoms

5.6. Has your dog ever had any of the following skin symptoms and how severe were they?

	Never	Few times	Once in a month – once in a week	Once in a week – every second day	Couple times daily	Constantly
Dog rubs or scratches its snout						
Dog scratches, rubs or licks its corner of the mouth						
Dog scratches or rubs its eyes						
Dog scratches or rubs its ears						
Dog scratches, rubs or licks its feet						
Dog scratches, rubs, eats or licks its groin or inguinal areas						
Dog licks, bites or rubs its caudal back						
Dog scratches, rubs, eats or licks itself all over						

5.7. Has your dog ever had redness or eczema?

A. Not at all
B. Around the snout
C. At the corners of the mouth
D. Around the eyes

E. In the ears
F. Between the toes
G. Under the feet
H. On the abdomen
I. In joint folds
J. On other skin areas

5.8. Has your dog ever had any of the following symptoms..

	Not at all	Few times	Once in a month – once in a week	Once in a week – every second day	Couple times daily	Constantly/Many times in a day
sneezing?						
cough?						
running eyes?						
diarrhea?						
gas / flatulance?						
constipation?						
vomiting?						

5.9. Has your dog ever had (you can choose several options)..

A. Eye infections e.g. conjunctivitis
B. Urticaria (allergic reaction of skin that comes e.g. after an insect bite)
C. Recurring ear infections caused by bacteria or yeasts
D. Recurring skin infections caused by bacteria or yeasts
E. Recurring “hot spots”
F. Not easily cured skin infections
G. Skin turning dark
H. Alopecia (loosing hair coat) or thinning hair coat
I. Skin thickening (please specify below)

5.10. If your dog has experienced thickening of the skin, on what parts did it happen?

5.11. Has your dog suffered from other skin symptoms? Please specify their character and location.

5.12. How old was your dog when skin related symptoms started? E.g. 3 years or 7,5 months

5.13. My dog shows most of its symptoms..

	More	Less	as much as in any season	I don’t know
in summer				
in spring				
in autumn				
in winter				
in humid weather				

Allergic symptoms and diseases of the dog, Part 2/2

6.1. If you have treated the skin symptoms of your dog, please specify the result of it:

	Symptoms decreased	Symptoms increased	No effect	I don't know	I have not used this treatment
Antibiotics					
Antihistamine					
Cyclosporine					
Immunotherapy					
Fatty acid product					
Local corticosteroid					
Systemic corticosteroid (taken perorally)					
Shampoo					
Diet					
Other treatment					
Skin cream					
Ear drops					

6.2. Has your dog been on an elimination diet?

1. No
2. Yes (specify below)

6.3. What happened during the elimination diet of your dog?

6.4. How the elimination diet affected on the symptoms of your dog (you can choose several options)?

A. There was no change in symptoms
B. The symptoms eased
C. The symptoms worsened
D. When the dog returned to its basic diet there was no change in symptoms
E. When the dog returned to its basic diet the symptoms eased
F. When the dog returned to its basic diet the symptoms worsened
G. The elimination diet was kept

6.5. If your dog has been treated with something else, describe?

6.6. If your dog has been allergy tested, please mark the used test and the results:

6.7. Please mark if the near family of your dog has suffered from skin symptoms

	I do not know	These relatives does not exist	No symptoms	Allergic/Atopic disease	Other skin disease	Both Allergic/Atopic disease and other skin disease
Dog's parents						

Dog's siblings						
Other relatives						

6.8. If you have other dogs or animals in the house, do they suffer from skin symptoms? If yes, what kind of skin symptoms?

6.9. Does your dog suffer from any chronic disease?

1. No
2. Yes

6.10. If yes, what disease?

6.11. Does dog have a permanent medication, if yes what kind of?

6.12. People currently living in your household: list the birth years of these people (e.g. 1957, 1954, and 1986):

6.13. Do any of the adult humans living with the dog have any of the following diseases?

	Yes, according to own judgement	Yes, diagnosed by medical doctor	No
Allergic rhinitis			
Atopic eczema			
Asthma			

6.14. Do any of the children living with the dog have any of the following diseases?

	Yes, according to own judgement	Yes, diagnosed by medical doctor	No
Allergic rhinitis			
Atopic eczema			
Asthma			

<u>Dog's diet</u>

7.1. My dog eats mainly

1. Manufactured dog food, that is from pet shop
2. Manufactured dog food, that is from market
3. Home food made specially for dog
4. Leftovers from the food of the humans
5. Raw food (BARF)
6. Both manufactured and raw food
7. Both raw food and home food
8. Manufactured dog food, that is from pharmacy or veterinary shop
9. Something else (specify below):

7.2. If your dog eats something else, what is it?

7.3. Does your dog get additional nutrients (e.g. vitamins or oils)?

1. Basically never
2. Sometimes
3. Regularly

7.4. If your dog gets additional nutrients, write down the product name:

<u>Dog's outdoor activity</u>

8.1. How often is your dog outdoors or go for a walk in a day?

1. Dog goes for a walk thrice or often in a day
2. Dog goes for a walk twice in a day
3. Dog goes for a walk once in a day
4. Dog is outside all the time or part of the time (e.g.in dog yard) and goes also for a walk daily
5. Dog is outside all the time or part of the time (e.g.in dog yard) and goes also for a walk few times a week
6. Dog is outside all the time or part of the time (e.g.in dog yard) and goes also for a walk less than once in a week
7. Dog is outdoors and/or goes for a walk other ways (please specify below):

8.2. If your dog is outdoors and/or goes for a walk other ways, tell how?

8.3. How much dog is walking daily?

1. 3 hours or more
2. 2 hours
3. an hour
4. about half an hour
5. less than half an hour

8.4. During the walk is your dog..

1. free all the time or most of the time
2. free part of time and other part in leashed
3. almost always in leashed
4. always in leashed

8.5. In what kind of environment do you mainly walk with the dog?

1. In forest using trails or terrain
2. In forest using tracks or other outdoor routes
3. In field using trails or terrain
4. In field using street
5. In parks of Conurbation area using sand or asphalt streets
6. In urban areas Conurbation area using asphalt streets
7. In dog parks

8.6. Do you let your dog or does your dog do following things (you can choose several options):

A. Eats soil, clay or stones
B. Drinks water from puddles, ditches or other drainage systems
C. Carries or gnaws sticks
D. Eats grass or other vegetation

E. Eats or licks faeces or urine

Dog's home and environment

9.1. The dog's current home is:

1. In city center
2. In municipality center
3. In Conurbation of urban area (suburban)
4. In Conurbation of country-side
5. In sparsely populated area

9.2. The dog lives mainly:

1. Inside
2. Outside chained
3. Outside in dog yard
4. Partly outside and partly inside
5. Other (specify below):

9.3. If your dog lives other, describe how:

9.4. If your dog lives inside, what kind of residence does it live?

1. In flat/apartment
2. In row house
3. In town house
4. In farm house

9.5. Is there agricultural activities in your dog's current home (your home)?

1. No
2. Yes, a part-time
3. Yes, a full-time

9.6. The household also includes (you can choose several options):

A. Other dog or dogs
B. Cat or cats
C. Other indoor pets with fur
D. Other outdoor pets
E. Farm animals
F. Furless indoor pets

9.7. How many dogs have you owned during your adult life (including the current dog)?

9.8. How long in average is your dog daily without presence of humans?

1. 0 hours
2. 1-2 hours
3. 3-6 hours
4. 7-8 hours
5. 9-10 hours
6. over 10 hours

9.9. Does someone smoke inside in places where your dog spends a lot of time?

1. Yes, often
2. Yes, sometimes
3. Yes, seldom
4. Never

9.10. Is your dog exposed to cigarette smoke while being outside (e.g. does someone smoke while walking with the dog)?

1. Yes, often
2. Yes, sometimes
3. Yes, but seldomly
4. Never

9.11. Does your dog spend time yearly in countryside (e.g. in summer cottage or with friends/relatives) for three weeks or more?

1. Yes
2. No

9.12. How far the nearest heavy industry locates from dog's current home?

1. Under 100 metres
2. 100-500 metres
3. About 1 kilometer distance
4. 2-5 kilometers
5. Further away
6. I do not know

9.13. How far the nearest farm locates from dog's current home?

1. Under 100 metres
2. 100-500 metres
3. About 1 kilometer distance
4. 2-5 kilometers
5. Further away
6. I do not know

9.14. If you want to specify something in previous questions, you can write here:

9.15. If you want, you can give feedback here. Tell for example if you found some questions especially difficult:

Thank you for filling the questionnaire!
